# Subjective cognitive decline and cognitive change among diverse middle‐aged and older Hispanic/Latino adults: Results from the Study of Latinos–Investigation of Neurocognitive Aging (SOL‐INCA)

**DOI:** 10.1002/alz.14232

**Published:** 2024-09-05

**Authors:** Freddie Márquez, Wassim Tarraf, Sayaka Kuwayama, Deisha F. Valencia, Ariana M. Stickel, Alejandra Morlett‐Paredes, Lourdes R. Guerrero, Krista M. Perreira, Sylvia Wassertheil‐Smoller, Sara Gonzalez, Christian R. Salazar, Martha L. Daviglus, Linda C. Gallo, Hector M. González

**Affiliations:** ^1^ Department of Neurosciences University of California San Diego La Jolla California USA; ^2^ Institute of Gerontology & Department of Healthcare Sciences Wayne State University Detroit Michigan USA; ^3^ Department of Psychology San Diego State University San Diego California USA; ^4^ Gillings School of Global Public Health University of North Carolina Chapel Hill North Carolina USA; ^5^ Department of Epidemiology and Population Health Albert Einstein College of Medicine New York New York USA; ^6^ Institute for Memory Impairments and Neurological Disorders University of California Irvine Irvine California USA; ^7^ Institute for Minority Health Research University of Illinois at Chicago College of Medicine Chicago Illinois USA

**Keywords:** Alzheimer's disease, cognitive concern, cognitive decline, cognitive function, dementia, epidemiology, Hispanics, Hispanics/Latinos, Latinos, neuroepidemiology, neuropsychology, population neuroscience, subjective cognitive decline

## Abstract

**INTRODUCTION:**

The potential utility of subjective cognitive decline (SCD) as an early risk marker of Alzheimer's disease and related dementias is under consideration. We examined associations between SCD and cognitive change among middle‐aged and older Hispanic/Latino adults living in the United States.

**METHODS:**

The short‐form Everyday Cognition Scale (ECog‐12) was assessed to generate global, executive function, and memory‐related SCD scores. We used survey generalized regressions to model the change in learning, memory, verbal fluency, executive function, and global cognitive performance over 7 years as a function of SCD (at Visit 2).

**RESULTS:**

The mean age was 56.37 ± 8.10 years at Visit 1 (*n* = 6225). Higher ECog‐12 was associated with greater decline in global cognitive performance (ECog‐12 global: B = –0.17, standard error [SE] = 0.02; ECog‐12 executive: B = –0.15, SE = 0.02; ECog‐12 memory: B = –0.14, SE = 0.02, *p*’s < 0.001).

**DISCUSSION:**

These results support the link between subjective reports of cognitive decline and objectively measured 7‐year cognitive decline in community‐dwelling, middle‐aged, and older Hispanic/Latino adults.

**Highlights:**

- We found that nearly two‐thirds of diverse middle‐aged and older Hispanics/Latinos reported cognitive concerns in a large and representative population study.
- Self‐reported subjective experiences of cognitive decline reflect objective cognitive decline in US Hispanics/Latinos.
- The relationship is stronger among men compared to women.
- The relationship between subjective and objective changes to memory are stronger in those with cognitive concerns, and remain even in cognitively healthy individuals.

## 1 BACKGROUND

There is a need to identify early and pre‐symptomatic stages of Alzheimer's disease and related dementia (ADRD).^1^ This includes finding early and affordable population‐level risk markers to inform risk reduction, prevention, and treatment efforts. This process all begins with individuals recognizing and reporting their subjective experiences of cognitive decline to their family members and health‐care providers. Moreover, the practical utility and value of subjective cognitive decline (SCD) as an early risk marker of ADRD is what patients already discuss with their health‐care providers. Elevating appreciation of the utility and value of SCD for risk‐stratifying individuals does face challenges in a field in which exciting new technologies garner the most clinical and scientific interest.

SCD is defined as a perceived persistent decline in cognition compared to a previously normal status, reported by the participant or an informant, not explained by mild cognitive impairment (MCI) or Alzheimer's disease (AD).^2^ Previous studies have found that cognitively unimpaired individuals reporting SCD have higher rates of progression to MCI and dementia and experience a faster decline compared to those without self‐reported SCD.^3^ Moreover, SCD is increasingly recognized as an early marker of cognitive decline.^4^, ^5^

Some of the gaps in the scientific literature currently include methodological challenges that limit the generalizability of previous findings. Notably, the operationalization and measurement of SCD are inconsistent, as an SCD definition has not been universally implemented, and the assessments are often brief, asking a single question.^2^ For instance, the Behavioral Risk Factor Surveillance System (BRFSS) survey study estimated that the overall prevalence of SCD in the United States is 11.3% for adults aged ≥ 45 years in 2019 when asking respondents whether they had experienced confusion or memory loss that is happening more often or is getting worse during the past 12 months.^6^ Incorporating cognitive domain‐specific SCD measures, like the Everyday Cognition Scale (ECog), may offer more precise identification of potential cognitive phenotypes that could complement sensitive biomarkers in informing ADRD prevention and treatment options.

Another limitation in the literature is the populations being studied. Currently, there is a gap in the scientific literature regarding early risk factors for ADRD among Hispanic/Latino adults who constitute the largest racial or ethnic minority in the United States. In a study with a sample of cognitively unimpaired and mildly cognitively impaired older adults, self‐reported, but not informant‐reported SCD, was related to objective cognitive performance among Hispanic/Latino adults.^7^ Additionally, in the Study of Latinos–Investigation of Neurocognitive Aging (SOL‐INCA) higher self‐reported SCD was associated with lower global cognitive, memory, and executive function scores in a cross‐sectional study of diverse representative Hispanic/Latino middle‐aged and older adults.^8^ However, it is unclear if SCD corresponds to objective cognitive decline over time in this population.

In this study, we examined associations between SCD and objective cognitive change among diverse Hispanic/Latino middle‐aged and older adults living in the United States. We hypothesized that higher SCD would be associated with greater adverse change in global, and domain‐specific (memory and executive function), cognitive scores. Moreover, the association between SCD and objective cognitive change in midlife has not been well described, and there is a paucity of research examining sex differences in SCD, but some recent evidence has suggested sex differences in AD risk states such as SCD.^9^, ^10^ Therefore, we then examined interaction effects of the exposures by age and sex.

## 2 METHODS

### 2.1 Population

The Hispanic Community Health Study/Study of Latinos (HCHS/SOL) is a prospective cohort study of *N* = 16,415 self‐identified Hispanic/Latinos (ages 18–74). It used a complex sampling design, which includes stratification and clustering, for its probability‐based sample (Visit 1; 2008–2011).^11^ The complex survey sampling procedures used in HCHS/SOL were designed to yield representative data for Hispanics/Latinos in four targeted US metropolitan areas: Bronx, NY; Chicago, IL; Miami, FL; and San Diego, CA. Each Field Center enrolled ≈ 4000 eligible, self‐identified Hispanics/Latinos from diverse backgrounds. At Visit 1, a two‐stage area probability sample of households was selected with stratification and oversampling incorporated at each stage to provide a broadly diverse sample, offer efficiencies in field operations, and ensure that the target age distribution is obtained. SOL‐INCA, an ancillary study of the HCHS/SOL conducted during the second HCHS/SOL visit, examines neurocognition among a subset of participants from the HCHS/SOL. Those on active military service, not currently living at home, planning to move from the area in the next 6 months, or unable to attend the in‐person clinic examination were excluded.^11^, ^12^ Inclusion criteria were: (1) Visit 2 completion, (2) Visit 1 neurocognitive testing completion, and (3) age ≥ 50 years at Visit 2. Of this group 222 were determined to be ineligible (e.g., missing Visit 1 data), 569 were eligible but refused, and 6377 were eligible and agreed to participate, and completed the SOL‐INCA visit. All evaluations and cognitive testing were conducted in the participant's preferred language (English or Spanish) by trained bilingual field center staff. Eligible participants returning for the SOL‐INCA visit had largely similar Visit 1 characteristics compared to those in the overall Visit 1 eligible participant pool. Furthermore, to guard against possible biases by sample attrition, the HCHS/SOL Coordinating Center generated study‐specific calibrated probability weights that adjust for non‐response (e.g., deaths) and allow generalization of estimates to the HCHS/SOL metropolitan area target populations aged ≥ 50 years. For this study, we excluded *n* = 152 observations with missing data on any of the model exposures or covariates as specified below for a final unweighted analytical sample of *n* = 6225. All participants provided informed consent, and the study protocol was approved by institutional review boards at all participating institutions.

**RESEARCH IN CONTEXT**

1. **Systematic review**: The authors reviewed the literature using electronic databases (e.g., PubMed) and search engines (e.g., Google Scholar). Previous publications showed that Hispanics/Latinos are at higher risk of Alzheimer's disease (AD) compared to non‐Hispanic Whites, but the research on subjective cognitive decline (SCD) among Hispanics/Latinos is limited, and its relationship with cognition warranted further investigation. We cite the overall findings on SCD and cognition, and the limited literature among Hispanics/Latinos.
2. **Interpretation**: Our findings suggest that approximately two thirds of middle‐aged and older Hispanics/Latinos reported cognitive concerns. SCD may be an indicator of objective cognitive decline among diverse middle‐aged and older community‐dwelling Hispanics/Latinos. These effects were modified by sex, and stronger in men than women. The relationship between subjective and objective memory‐related changes was particularly stronger among those with cognitive concerns, and remains significant in cognitively unimpaired individuals.
3. **Future directions**: The relationship between SCD and measures of AD pathology (e.g., amyloid beta and tau), neural correlates, future decline, dyadic SCD assessments from participants and informants, and longitudinal prospective measures, which may provide additional information about disease stage and progression in preclinical and prodromal AD, should be investigated in the future.

### 2.2 Cognitive testing

At the HCHS/SOL visit (Visit 1), the Neurocognitive Reading Center trained and the Field Centers directly supervised bicultural/bilingual technicians who administered the brief cognitive battery, which included three tests: the Brief‐Spanish English Verbal Learning (B‐SEVLT),^13^ Word Fluency (WF), and Digit Symbol Subtest (DSS)^14^ tests. These three tests provided four scores: (1) the B‐SEVLT Sum score for verbal episodic learning (the summed total of correctly learned items across three trials [range, 0–45]); (2) the B‐SEVLT Recall score for verbal episodic memory (total correctly recalled items after an interference trial; range, 0–15); (3) the WF score on a phonemic verbal fluency test (sum of correctly generated words within 1 minute for the letters F and A; range, 0–50); and (4) the DSS score on a mental processing speed and executive functioning examination. A global cognitive composite score (global cognition) was derived by averaging the *z* scores across the four domain‐specific scores, as described herein. Additional information about the cognitive tests assessed at Visit 1 and the cohort has been previously published.^15^ The SOL‐INCA visit (Visit 2) repeated cognitive tests administered at Visit 1 for eligible HCHS/SOL participants who returned for Visit 2 with a mean follow‐up of ≈ 7 years. Similar to cognitive tests at Visit 1, a global cognitive composite score was generated by averaging the z scored domain‐specific test scores. At the SOL‐INCA visit, the cognitive battery additionally included Trail‐Making Tests Parts A and B (TMT‐A and ‐B; processing speed and executive functioning), and the brief self‐reported SCD questionnaire (ECog‐12). More detailed information about the battery of tests has been previously published.^12^, ^16^ Cognitive change scores for repeated cognitive tests at Visits 1 and 2 as well as global cognitive composite were calculated using regression‐based methods. Weighted linear regression models were used to predict cognitive performance at Visit 2 (SOL‐INCA) as a function of Visit 1 cognitive performance, adjusting for elapsed time (in days) between cognitive assessments. Regression‐based change score methods and their application to neurocognitive measures have been detailed elsewhere.^17^ This method yields a single measure depicting cognitive change that can be modeled subsequently using standard regression techniques. Test‐specific as well as global standardized measures of change were calculated using (*T2 – T2_pred_)/RMSE*, where *T2* represents a respondent's score on a cognitive test at Visit 2, *T2_pred_* is the predicted score, and *RMSE* is root mean squared error of the fitted model. Global and test‐specific measures of objective cognitive change were modeled using survey regression analyses as described in section 2.6.

### 2.3 Subjective cognitive decline

SCD was measured at Visit 2 with the short (12‐item) form of the ECog (ECog‐12), which was developed as an informant‐rated report of cognitively mediated functional abilities in older adults.^18^, ^19^ Although we used the self‐report version of the ECog‐12 in this study, as opposed to the informant version, the self‐report version has been shown to predict progression to MCI.^20^ Previous research suggests that the ECog has good psychometric properties,^18^ and the short form used in this study discriminates between dementia and normal cognition.^19^ Moreover, the ECog has been used in several studies as a measure of SCD.^21^, ^22^, ^23^, ^24^ A recent study found that the cross‐sectional relationship between informant‐reported ECog scores and neuropsychological test performance was very similar across a group of non‐Hispanic White, Black, and Hispanic/Latino individuals.^25^ The ECog‐12 asks participants to rate their current ability to perform cognitively mediated daily tasks related to everyday memory, language, visuospatial abilities, and executive functions compared to their ability to do the same task 10 years ago. Items are rated on a scale of 1 to 4, with 1 = better or no change and 4 = consistently much worse. For our analyses, the global as well as executive and memory subdomain SCD exposures (i.e., ECog‐12 global, ECog‐12 executive, ECog‐12 memory) were generated by averaging the corresponding component item scores. Finally, all ECog‐12 exposure variables (global, executive, and memory) were *z* scored ([Xi‐Mean]/standard deviation) using the derived means and standard deviations of the SOL‐INCA target population to facilitate comparisons of results across models. Cognitive concern (or worry) was also measured based on responding Yes to the question: Are you worried or believe that you are having problems with your attention, concentration, or memory? (No, Yes). Concerns (or worry) about cognitive decline can be associated with different objective levels of cognitive and functional impairment. There is also evidence that concerns (worries) increase likelihood of cognitive decline or conversion to dementia.^26^ Thus, cognitive concern (worry) is included as a feature that increases the risk of cognitive decline in a concept called SCD plus.^2^, ^27^

### 2.4 Covariables

All covariables were measured at Visit 1. Covariables included age in years (< 60; 60–69; and ≥ 70), sex (female, male), level of education (less than high school, high school or equivalent, more than high school), and Hispanic/Latino background (Dominican, Central American, Cuban, Mexican, Puerto Rican, South American, more than one/other). Given the extensive literature linking cardiovascular disease (CVD) risk factors to cognitive decline in Hispanic/Latino adults,^28^, ^29^, ^30^, ^31^ we also included Framingham CVD 10‐year risk score. Finally, we also adjusted for residual effects of variations in depression and anxiety symptoms by controlling for the Center for Epidemiologic Studies Depression Scale‐10 (CESD‐10),^32^ and the State‐Trait Anxiety Inventory (STAI) scores, respectively.

### 2.5 Modifiers

Additionally, we examined potential modifiers including age and sex. The prevalence of SCD is similar in midlife compared to older adulthood,^33^ yet its association with objective cognition at midlife has not been well described. Furthermore, while there are numerous findings revealing sex differences in the prevalence of AD, there is a paucity of research examining sex differences in SCD. Some recent evidence has suggested sex differences in AD risk states such as SCD.^9^, ^10^

### 2.6 Analytic approach

#### 2.6.1 Statistical analyses

First, we generated descriptive statistics to characterize our target population by cognitive concern groups. Differences between the cognitive concern groups were tested using survey‐adjusted chi‐squared tests for categorical variables and Wald tests for continuous variables. The survey‐weighted estimates are presented in Table 1. Weighted mean scores of neurocognitive tests at Visit 1 and Visit 2 as well as the cognitive change scores are presented in Table S1 in supporting information. Group differences in these scores by cognitive concern groups were tested using survey‐adjusted Wald tests.

**TABLE 1:** Descriptive statistics to characterize the population of SOL‐INCA overall and across cognitive concern subgroups.

	No cognitive concern	Cognitive concern	Overall	
	(*N* = 2048)	(*N* = 4177)	(*N* = 6225)	*p* value
**Age (%, SE)**				
< 60 Years	68.54 (1.52)	63.56 (1.33)	65.22 (1.04)	**0.027**
60–69 Years	25.80 (1.45)	28.50 (1.17)	27.59 (0.93)	
70+ Years	5.66 (0.84)	7.95 (0.76)	7.18 (0.59)	
**Sex (%, SE)**				
Female	46.96 (1.49)	58.61 (1.05)	54.71 (0.84)	**<0.001**
Male	53.04 (1.49)	41.39 (1.05)	45.29 (0.84)	
**Education (%, SE)**				
Less than HS	32.28 (1.57)	41.37 (1.26)	38.33 (1.06)	**<0.001**
HS or equivalent	20.33 (1.24)	21.46 (0.95)	21.08 (0.75)	
More than HS	47.39 (1.46)	37.17 (1.20)	40.60 (1.00)	
**Hispanic/Latino background (%, SE)**				
Dominican	11.04 (1.11)	8.59 (0.80)	9.41 (0.77)	**0.025**
Central American	7.15 (0.82)	7.24 (0.66)	7.21 (0.56)	
Cuban	24.39 (2.17)	26.21 (2.04)	25.60 (1.89)	
Mexican	32.22 (2.04)	33.95 (1.82)	33.37 (1.69)	
Puerto‐Rican	14.64 (1.03)	15.77 (0.97)	15.39 (0.83)	
South American	5.22 (0.56)	5.12 (0.45)	5.16 (0.38)	
More than one/Other	5.33 (0.95)	3.11 (0.47)	3.85 (0.45)	
**Age (mean, SD)**	55.63 (7.66)	56.75 (8.30)	56.37 (8.10)	**0.002**
**Framingham CVD 10‐year risk (mean, SD)**	15.90 (13.19)	16.03 (13.72)	15.99 (13.54)	0.815
**CESD‐10 Depressive symptoms (mean, SD)**	5.49 (5.10)	8.31 (6.58)	7.37 (6.26)	**<0.001**
**10‐Item State Trait Anxiety Inventory (mean, SD)**	15.02 (4.78)	17.76 (6.05)	16.84 (5.80)	**<0.001**

*Note*: Results are derived from chi‐square tests and Wald tests using data from the Study of Latinos–Investigation of Neurocognitive Aging (SOL‐INCA unweighted *n* = 6225). Sample size is unweighted; all other reported values are weighted. All variables are measured at Visit 1. Bold values denote statistical significance.

Abbreviations: CESD, Center for Epidemiological Studies‐Depression; CVD, cardiovascular disease; HS, high school; SD, standard deviation; SE, standard error.

**FIGURE 1:**
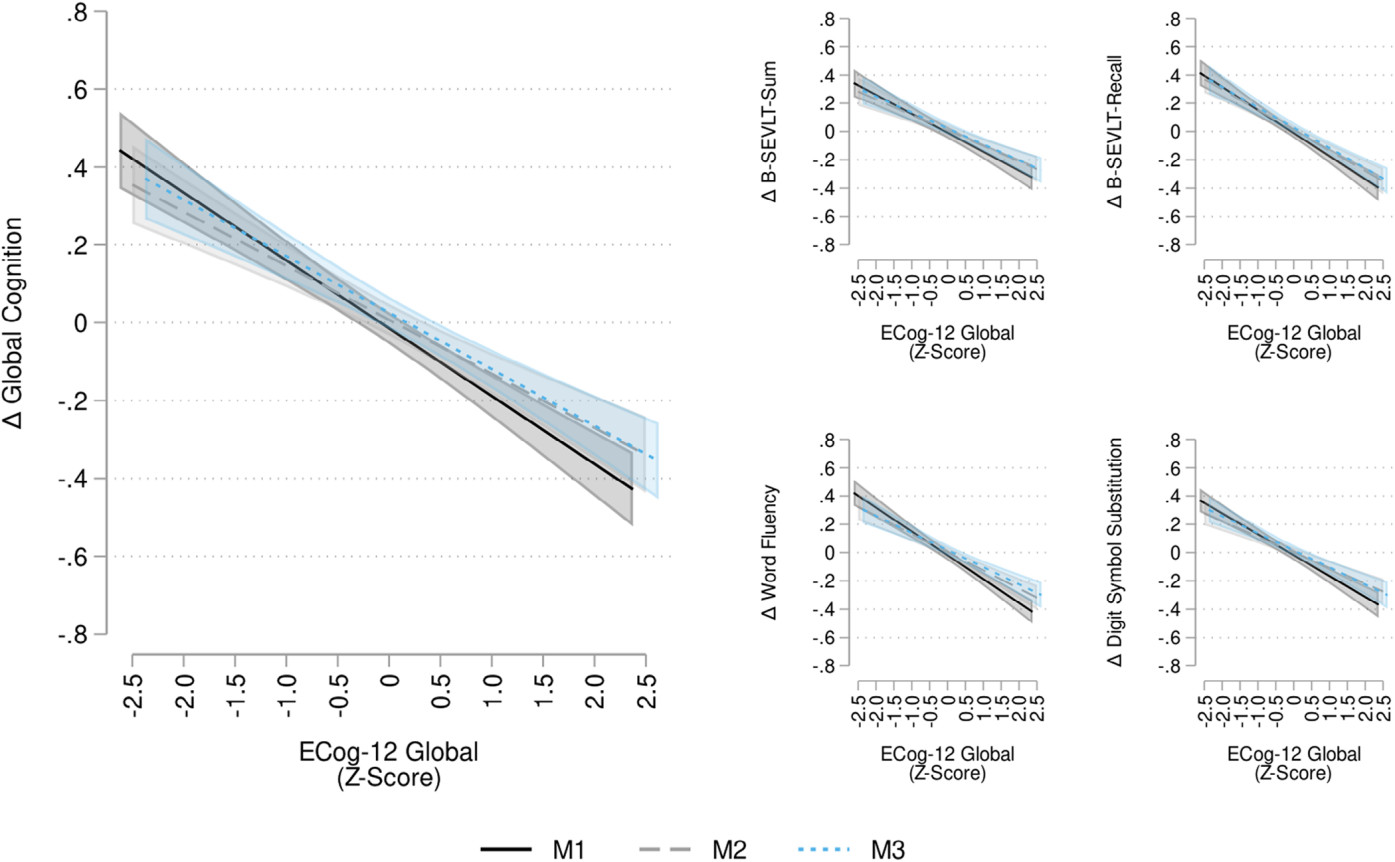
Association between ECog‐12 global and change in cognitive performance. Results are derived from survey linear regression models using data from the Study of Latinos–Investigation of Neurocognitive Aging (SOL‐INCA unweighted *n* = 6225); Δ, change; M1 is an unadjusted model; M2 is adjusted for age, sex, education, and Hispanic/Latino background; M3 is additionally adjusted for Framingham Cardiovascular Disease 10‐year risk score, Center for Epidemiological Studies‐Depression—Depressive symptoms, and 10‐Item State Trait Anxiety Inventory. B‐SEVLT, Brief Spanish English Verbal Learning Test; ECog‐12, 12‐Item form of the Everyday Cognition Scale; Global, Global Cognition.

Second, to examine the hypothesized associations between the ECog‐12 exposures and cognitive change outcomes, as generated from the regression‐based methods described above, we fit a series of survey generalized regression models sequentially adjusting for covariables. We tested (1) unadjusted; (2) adjusted for age, sex, education, and Hispanic/Latino background; and (3) included the Framingham CVD‐10 year risk score, depression (using the CESD‐10) and state anxiety scores (based on the STAI). The estimated coefficients (β) and their standard errors are presented in Table 2. In post hoc analyses, we calculated average marginal estimates and plotted these with their 95% confidence intervals to facilitate interpretation (Figure 1; Figures S1–S2 in supporting information).

**TABLE 2:** Association between subjective cognitive decline scores (ECog‐12) and change in cognitive performance in the overall SOL‐INCA population.

	Δ B‐SEVLT Sum			Δ B‐SEVLT Recall			Δ Word fluency			Δ Digit Symbol Substitution			Δ Global cognition		
	M1	M2	M3	M1	M2	M3	M1	M2	M3	M1	M2	M3	M1	M2	M3
ECog‐12 global	−0.13***	−0.11***	−0.11***	−0.16***	−0.14***	−0.14***	−0.17***	−0.13***	−0.12***	−0.15***	−0.11***	−0.12***	−0.17***	−0.14***	−0.15***
	(0.02)	(0.02)	(0.02)	(0.02)	(0.02)	(0.02)	(0.02)	(0.02)	(0.02)	(0.02)	(0.02)	(0.02)	(0.02)	(0.02)	(0.02)
ECog‐12 executive	−0.12***	−0.09***	−0.09***	−0.14***	−0.12***	−0.11***	−0.15***	−0.11***	−0.10***	−0.14***	−0.10***	−0.11***	−0.15***	−0.12***	−0.12***
	(0.02)	(0.02)	(0.02)	(0.02)	(0.02)	(0.02)	(0.02)	(0.02)	(0.02)	(0.02)	(0.02)	(0.02)	(0.02)	(0.02)	(0.02)
ECog‐12 memory	−0.12***	−0.09***	−0.09***	−0.14***	−0.12***	−0.12***	−0.11***	−0.08***	−0.07***	−0.12***	−0.09***	−0.09***	−0.14***	−0.11***	−0.11***
	(0.02)	(0.02)	(0.02)	(0.02)	(0.02)	(0.02)	(0.02)	(0.02)	(0.02)	(0.02)	(0.02)	(0.02)	(0.02)	(0.02)	(0.02)

*Note*: Results are derived from survey linear regression models using data from the Study of Latinos–Investigation of Neurocognitive Aging (SOL‐INCA unweighted *n* = 6225). All variables are *z* scored. M1 is a unadjusted model; M2 is adjusted for age, sex, education, and Hispanic/Latino background; M3 is additionally adjusted for Framingham Cardiovascular Disease 10‐year risk score, Center for Epidemiological Studies‐Depression—Depressive Symptoms, and 10‐Item State Trait Anxiety Inventory.

Abbreviations: B‐SEVLT, Brief Spanish English Verbal Learning Test; ECog‐12, 12‐item form of the Everyday Cognition Scale; Executive, executive function; Global, global cognition; Δ, change.

*p < 0.05.

**p < 0.01.

*** *p* < 0.001.

**FIGURE 2 alz14232-fig-0002:**
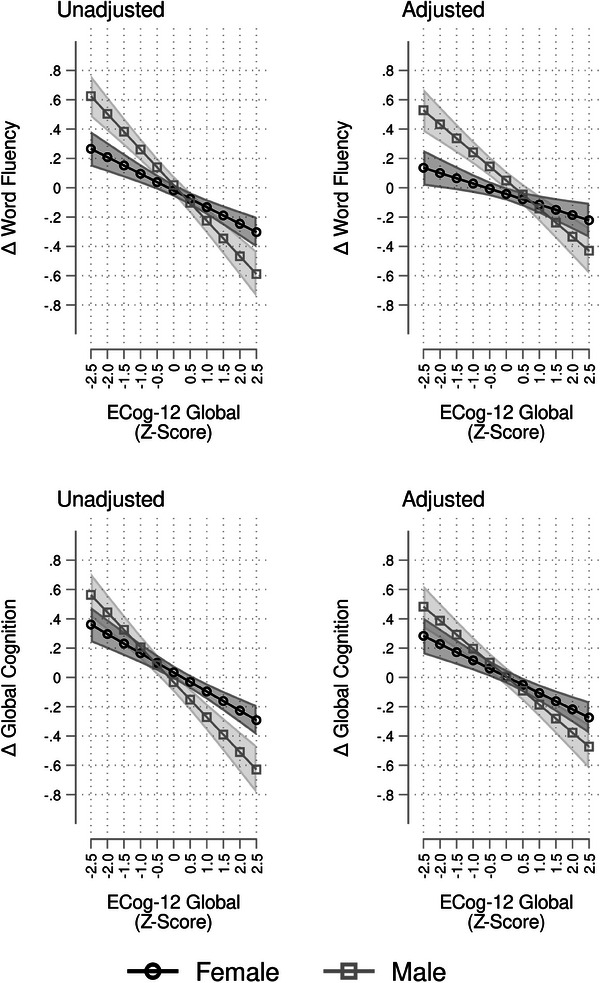
Associations between ECog‐12 global and change in cognitive performance, modified by sex. Results are derived from survey linear regression models using data from the Study of Latinos–Investigation of Neurocognitive Aging (SOL‐INCA unweighted *n* = 6225); Δ, change; adjusted models include the following covariates: age, sex, education, Hispanic/Latino background, Framingham Cardiovascular Disease 10‐year risk score, Center for Epidemiological Studies‐Depression—Depressive symptoms, and 10‐Item State Trait Anxiety Inventory. ECog‐12, 12‐Item form of the Everyday Cognition Scale.

**FIGURE 3 alz14232-fig-0003:**
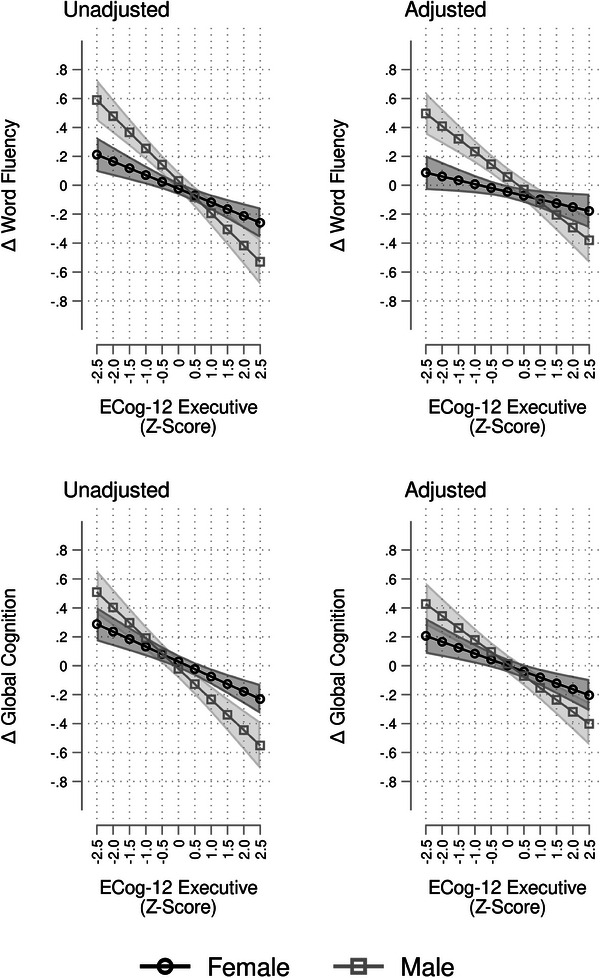
Associations between ECog‐12 executive and change in cognitive performance, modified by sex. Results are derived from survey linear regression models using data from the Study of Latinos–Investigation of Neurocognitive Aging (SOL‐INCA unweighted *n* = 6225); Δ, change; adjusted models include the following covariates: age, sex, education, Hispanic/Latino background, Framingham Cardiovascular Disease 10‐year risk score, Center for Epidemiological Studies‐Depression—Depressive symptoms, and 10‐Item State Trait Anxiety Inventory. ECog‐12, 12‐Item form of the Everyday Cognition Scale; Executive, Executive Function.

Third, we tested interaction effects of the exposures by age and sex, using the same sequence of model adjustments specified above. The test statistics are presented in Table 3. For the significant interactions, we calculated and plotted the marginal means and their 95% confidence intervals to illustrate the differential associations of our outcomes and exposures by levels of the modifiers (Figures 2, 3).

**TABLE 3 alz14232-tbl-0003:** Estimated interactions of the associations between subjective cognitive decline (ECog‐12) and change in cognitive performance by age, sex, and cognitive concern in the overall SOL‐INCA population.

(i) Age (df = 2)												
	ECog‐12 global				ECog‐12 executive				ECog‐12 memory			
	Unadjusted		Adjusted		Unadjusted		Adjusted		Unadjusted		Adjusted	
Cognitive change	F	*p* value	F	*p* value	F	*p* value	F	*p* value	F	*p* value	F	*p* value
**Δ B‐SEVLT Sum**	1.40	0.25	1.31	0.27	1.01	0.37	0.81	0.44	2.51	0.08	1.96	0.14
**Δ B‐SEVLT Recall**	0.09	0.91	0.02	0.98	0.24	0.79	0.01	0.99	0.86	0.42	0.31	0.74
**Δ WF**	1.69	0.19	1.59	0.20	0.78	0.46	0.74	0.48	1.78	0.17	1.32	0.27
**Δ DSS**	1.43	0.24	1.96	0.14	2.14	0.12	2.43	0.09	1.53	0.22	1.66	0.19
**Δ Global**	0.18	0.83	0.35	0.70	0.29	0.75	0.35	0.71	0.86	0.42	0.72	0.49

(ii) Sex (df = 1)												
**Δ B‐SEVLT Sum**	1.65	0.20	0.38	0.54	1.38	0.24	0.38	0.54	0.002	0.97	0.10	0.76
**Δ B‐SEVLT Recall**	3.61	0.06	1.63	0.20	3.59	0.06	2.06	0.15	2.13	0.14	0.95	0.33
**Δ WF**	12.51	**<0.001**	11.40	**0.001**	12.61	**<0.001**	11.86	**0.001**	2.10	0.15	2.19	0.14
**Δ DSS**	3.46	0.06	2.19	0.14	2.04	0.15	1.26	0.26	0.35	0.55	0.13	0.71
**Δ Global**	10.11	**0.002**	6.44	**0.01**	9.64	**0.002**	6.77	**0.01**	2.01	0.16	1.00	0.32

(iii) Cognitive concern (df = 1)												
**Δ B‐SEVLT Sum**	0.96	0.33	1.21	0.27	0.19	0.66	0.28	0.60	0.67	0.41	0.06	0.81
**Δ B‐SEVLT Recall**	1.01	0.31	1.05	0.31	1.24	0.27	1.31	0.25	8.64	**0.003**	6.55	**0.01**
**Δ WF**	0.002	0.97	0.11	0.74	0.01	0.93	0.06	0.81	1.74	0.19	1.73	0.19
**Δ DSS**	2.38	0.12	1.69	0.19	0.96	0.33	0.77	0.38	0.87	0.35	1.47	0.23
**Δ Global**	0.14	0.70	0.07	0.80	0.004	0.95	0.001	0.97	2.28	0.13	1.55	0.21

*Note*: Results are derived from survey linear regression models using data from the Study of Latinos–Investigation of Neurocognitive Aging (SOL‐INCA unweighted *n* = 6225). Adjusted models include the following covariates: age, sex, education, Hispanic/Latino background, Framingham Cardiovascular Disease 10‐year risk score, Center for Epidemiological Studies‐Depression—Depressive Symptoms, and 10‐Item State Trait Anxiety Inventory. Bold values denote statistical significance.

Abbreviations: B‐SEVLT,  Brief Spanish English Verbal Learning Test; Δ, change; df, degree of freedom; DSS, Digit Symbol Substitution; ECog‐12,  12‐item form of the Everyday Cognition Scale; Executive, executive function; F, f test; Global, global cognition; WF, Word Fluency.

The HCHS/SOL study designs and sampling procedures allow generalization of estimates to the HCHS/SOL metropolitan area target populations. Study‐specific calibrated probability weights adjust for non‐response (e.g., deaths) and allow generalization of estimates to the HCHS/SOL metropolitan area target populations aged ≥ 50 years. These probability weights were used to generate all estimates in the current study. All analyses were conducted in Stata 17 using the survey functionalities to appropriately account for the complex sampling design of HCHS/SOL and SOL‐INCA and allow generalizability to the SOL‐INCA target population.

#### 2.6.2 Sensitivity analyses

**FIGURE 4 alz14232-fig-0004:**
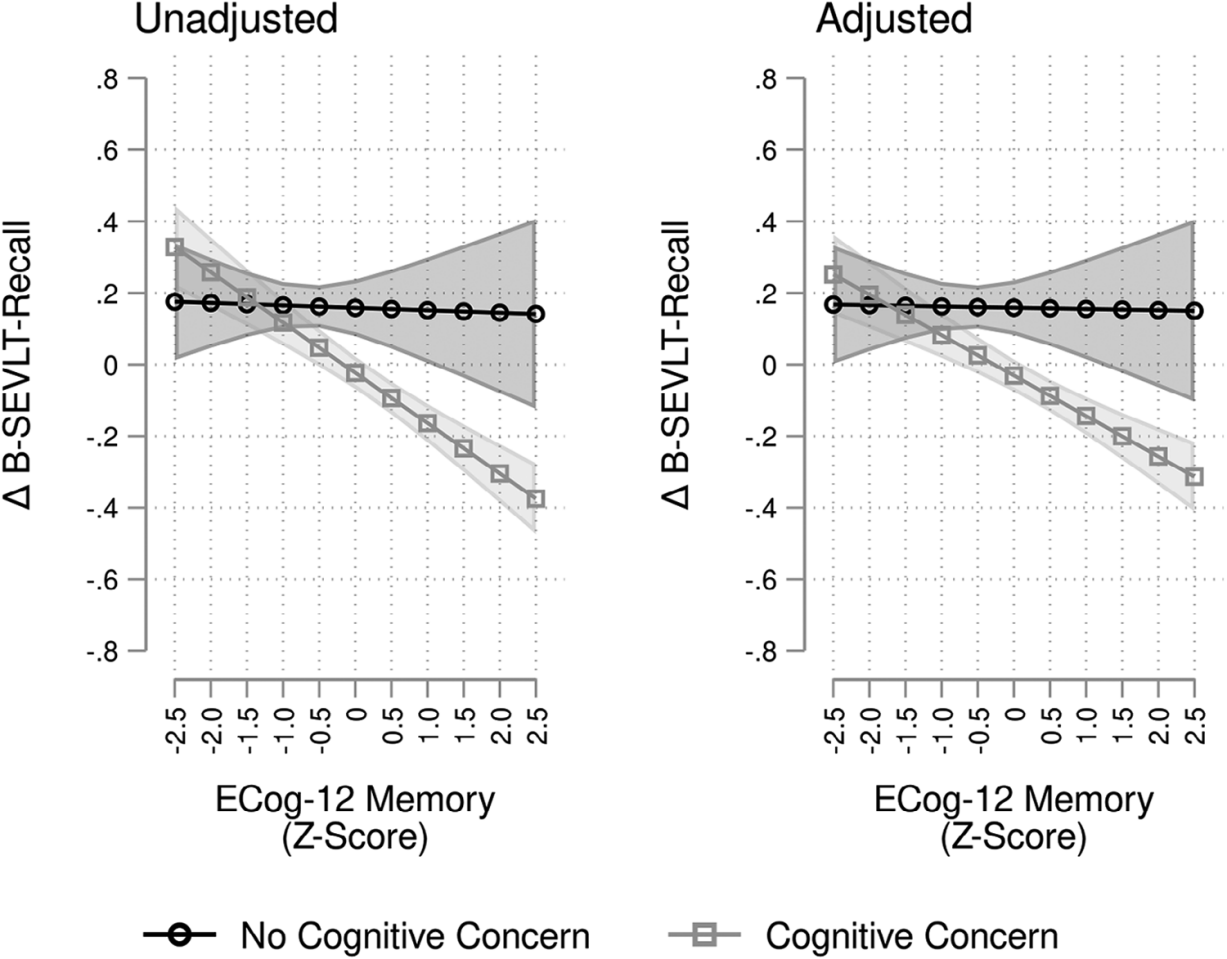
Associations between ECog‐12 memory and change in cognitive performance, modified by cognitive concern. Results are derived from survey linear regression models using data from the Study of Latinos–Investigation of Neurocognitive Aging (SOL‐INCA unweighted *n* = 6225); Δ, change; adjusted models include the following covariates: age, sex, education, Hispanic/Latino background, Framingham Cardiovascular Disease 10‐year risk score, Center for Epidemiological Studies‐Depression—Depressive symptoms, and 10‐Item State Trait Anxiety Inventory. B‐SEVLT, Brief Spanish English Verbal Learning Test; ECog‐12, 12‐Item form of the Everyday Cognition Scale.

We conducted two sets of sensitivity analyses to ensure that our results were not driven by individuals with MCI as well as individuals with concerns (or worry) about SCD. First, we examined the hypothesized associations between the ECog‐12 exposures and cognitive change outcomes using the cognitively unimpaired subpopulation by excluding individuals who met criteria for MCI (*n* = 582) or suspected severe impairment (*n *= 80). MCI (0 = No, 1 = Yes) was operationalized using National Institutes on Aging–Alzheimer's Association criteria as described in González et al.^16^ The descriptive statistics of the target population through this sensitivity are presented in Table S2 in supporting information, and the estimated regression coefficients and statistics (e.g., standard errors) are presented in Table 4. For the significant associations, we calculated and plotted the marginal means and the 95% confidence intervals to facilitate interpretation of the results (Figure S3 in supporting information). Second, we tested for effect modification of the association between ECog‐12 exposures and cognitive decline through self‐reported concerns (or worries) about SCD.^34^ The test statistics are presented in Table 3. For the significant interactions, we calculated and plotted the marginal means and their 95% confidence intervals to illustrate the differential associations of our outcomes and exposures by levels of the modifier (Figure 4).

**TABLE 4 alz14232-tbl-0004:** Association between ECog‐12 subjective cognitive decline and change in cognitive performance in the cognitively unimpaired subpopulation (excluding individuals with mild cognitive impairment or suspected severe impairment).

	Δ B‐SEVLT Sum			Δ B‐SEVLT Recall			Δ Word fluency			Δ DSS			Δ Global cognition		
	M1	M2	M3	M1	M2	M3	M1	M2	M3	M1	M2	M3	M1	M2	M3
ECog‐12 global	−0.01	0.01	0.01	−0.06**	−0.04*	**−0.05** *	−0.05**	−0.01	0.004	−0.01	0.02	0.01	−0.005	0.03	0.02
	(0.02)	(0.02)	(0.02)	(0.02)	(0.02)	**(0.02)**	(0.02)	(0.02)	(0.02)	(0.02)	(0.02)	(0.02)	(0.02)	(0.02)	(0.02)
ECog‐12 executive	−0.005	0.02	0.02	−0.04	−0.02	−0.02	−0.04*	−0.002	0.01	−0.02	0.01	0.003	0.01	0.04*	0.03
	(0.02)	(0.02)	(0.02)	(0.02)	(0.02)	(0.02)	(0.02)	(0.02)	(0.02)	(0.02)	(0.02)	(0.02)	(0.02)	(0.02)	(0.02)
ECog‐12 Memory	−0.01	0.01	0.01	−0.05**	−0.04*	**−0.04** *	−0.01	0.02	0.03	0.02	0.04*	0.03	0.01	0.04*	0.03
	(0.02)	(0.02)	(0.02)	(0.02)	(0.02)	**(0.02)**	(0.02)	(0.02)	(0.02)	(0.02)	(0.02)	(0.02)	(0.02)	(0.02)	(0.02)

*Note*: Results are derived from survey linear regression models using data from cognitively unimpaired subsample in the Study of Latinos–Investigation of Neurocognitive Aging (SOL‐INCA unweighted *n* = 5563). All variables are *z* scored. M1 is a crude model; M2 is adjusted for age, sex, education, and Hispanic/Latino background; M3 is additionally adjusted for Framingham Cardiovascular Disease 10‐year risk score, Center for Epidemiological Studies‐Depression—Depressive Symptoms, and 10‐Item State Trait Anxiety Inventory.

Abbreviations: B‐SEVLT,  Brief Spanish English Verbal Learning Test; Δ, change; DSS, Digit Symbol Substitution; ECog‐12,  12‐item form of the Everyday Cognition Scale; Executive, executive function; Global, global cognition.

* *p* < 0.05.

** *p* < 0.01

***p < 0.001.

Bold values denote statistical significance in M3.

## 3 RESULTS

### 3.1 Descriptive statistics

Table 1 displays the descriptive characteristics for the overall target population as well as by cognitive concern group (no cognitive concern vs. cognitive concern). The mean age was 56.4 years, nearly 55% were female, and > 60% had completed at least a high school education. Two thirds of the population had cognitive concerns. Comparing the groups reporting and not reporting cognitive concerns, those with cognitive concerns were more likely to be female, had lower educational attainment (specifically, they were less likely to have a high school degree), were older, and reported higher levels of depression and anxiety symptoms. We also observed significant differences based on Hispanic/Latino background.

### 3.2 Primary analysis

The estimated regression coefficients and inferential statistics from the models are presented in Table 2. All ECog‐12 exposures were associated with decline in global cognitive change (ECog‐12 global: B = −0.17, *p* < 0.001; ECog‐12 executive: B = −0.15, *p* < 0.001; ECog‐12 memory: B = −0.14, *p* < 0.001) in the unadjusted model. The associations were slightly attenuated, but they remained significant after adjustment for the covariates. All three ECog‐12 exposures were consistently associated with domain‐specific declines in learning and memory, word fluency, and processing speed/executive function scores. The estimated marginal means from the ECog‐12 global models are presented in Figure 1, ECog‐12 executive models are presented in Figure S1, and ECog‐12 memory models are presented in Figure S2.

### 3.3 Modification analysis

We found no evidence for significant effect modification by age of any associations of the ECog‐12 exposures with cognitive change outcomes.[Table alz14232-tbl-0003] The interaction between sex and ECog‐12 global was found to be significant in relationship to changes in word fluency (unadjusted *F*_1 _= 12.51, *p *< 0.001; Table 3) and global cognition (unadjusted *F*_1 _= 10.11, *p *= 0.002; Table 3), and more pronounced in males than females (Figure 2). The interaction between sex and ECog‐12 executive function was found to be significant in relation to changes in word fluency (unadjusted *F*_1 _= 12.61, *p *< 0.001; Table 3) and global cognition (unadjusted *F*_1 _= 9.64, *p *= 0.002; Table 3), and more pronounced in males than females (Figure 3).

### 3.4 Sensitivity analysis

The characteristics of the cognitively unimpaired participants, presented in the sensitivity analyses subpopulation (Table S2), were qualitatively similar to those of the original analytic population (presented in Table 1). Among individuals not meeting criteria for MCI or suspected severe impairment (cognitively unimpaired), we observed significant associations of ECog‐12 global and ECog‐12 memory with changes in memory scores (ECog‐12 global: B = −0.06, *p* < 0.01; ECog‐12 memory: B = −0.05, *p* < 0.01; Table 4). The estimated marginal means from the ECog‐12 global and ECog‐12 memory associations with change in memory scores are presented in Figure S3. These associations were not explained by adjustment for demographic characteristics, cardiovascular risk, and depression and anxiety symptoms. We also found significant associations of ECog‐12 global and ECog‐12 executive with decline in word fluency (ECog‐12 global: B = −0.05, *p* < 0.01; ECog‐12 executive: B = −0.04, *p* < 0.05), but these associations were fully explained by adjustment for demographic characteristics. Survey‐adjusted Wald tests showed significant group differences in weighted mean scores for neurocognitive tests at Visit 1, Visit 2, and the cognitive change scores by cognitive concern (Table S1). Furthermore, the interaction between cognitive concern and ECog‐12 memory was found to be significant in its relationship to changes in B‐SEVLT Recall (unadjusted *F*_1 _= 8.64, *p *= 0.003; Figure 4).

## 4 DISCUSSION

In this large, multi‐centered, community‐based population study of diverse Hispanic/Latino middle‐aged and older adults living in the United States, self‐reported subjective experiences of cognitive decline were associated with global and domain‐specific declines in cognitive performance. Nearly two thirds of participants reported concerns about attention, concentration, or memory. These self‐reported subjective experiences of cognitive decline, assessed by the ECog, were reflected in objectively measured declines in cognitive performance over 7 years. Moreover, higher subjective reporting of global cognitive decline was linked to greater declines in overall or global cognitive function. Similarly, higher subjective reports of memory decline were related to objectively measured memory decline. Additionally, higher subjective reports of executive function decline were related to objectively measured executive function decline. The results were only slightly attenuated after adjusting for cardiovascular disease risk, and mood symptoms (depression and state anxiety scores). The effects of subjective reports of global and executive decline on verbal fluency and global cognition were more pronounced in males compared to females. The relationship between subjective and objective memory‐related changes was more pronounced in those with cognitive concerns than those without. In cognitively unimpaired individuals, higher subjective reports of global and memory decline were related to objectively measured memory decline, but all other associations were attenuated. Our findings indicate that self‐reported experiences of SCD may improve the precision of risk stratifying the cognitive health of aging Hispanic/Latino adults.[Fig alz14232-fig-0002], [Fig alz14232-fig-0003], [Table alz14232-tbl-0004], [Fig alz14232-fig-0004]

The prevalence of ADRD is expected to nearly triple in coming decades, making it essential to identify early risk markers and screening tools for ADRD among the populations that are expected to face the burdens of ADRD. The Hispanic/Latino population currently constitutes the largest racial or ethnic minority in the United States and has a diverse background in terms of genetic ancestry, culture, and environmental exposures.^35^, ^36^ In coming decades, this population is projected to quadruple and have the largest increase in ADRD prevalence of any ethnic or racial group in the United States.^37^, ^38^ Investigating the relationship between SCD and cognition in the context of ADRD is especially important among Hispanic/Latino individuals, who face disparities in access to health care, are less likely to have a primary care provider, and are less likely to discuss memory concerns with a clinician.^39^ Our findings support that subjective assessment of cognitive decline is associated with objective cognitive decline in this population.

The association between SCD and cognitive decline was consistent across global, memory, and executive function domains. Our results were consistent with findings from the Vietnam Era Twin Study of Aging (VETSA), which reported modest associations between participant‐reported ECog scores and objective decline for memory, and executive function over 10 years among a community‐dwelling sample of adult male twins.^40^ Notably, sex differences have been reported in other studies.^9^, ^10^, ^41^ In the present study, we found that the associations between subjective experiences of global cognitive decline and change in global cognition were more pronounced in men compared to women. Subjective experiences of executive function decline and decline in objective verbal fluency performance also differed by sex. Verbal fluency is often described as an executive function, but it is also sometimes considered a measure of language ability. These results support that perceived cognitive dysfunction may manifest differently for women than men.

While most studies focus on older adulthood, midlife SCD warrants further investigation. In this study, participants expressing cognitive concern were older than those that did not. However, age did not modify the associations between SCD and change in cognitive performance. Recent studies suggest that SCD below age 60 may be related to subtle brain pathology.^42^ This would suggest that despite differences in cognitive concern among age groups, individuals’ reports of SCD are also important in midlife. From a public health perspective, it is perhaps more important to assess SCD in midlife to mitigate further declines in cognitive health.

Our study estimated a 67%, or two in three adults, prevalence of cognitive concern in the overall SOL‐INCA population. Associations between subjective memory decline and objective memory decline were more pronounced in individuals with concerns (or worries) about their attention, concentration, or memory compared to those with no concern. These results are consistent with recent findings.^43^, ^44^ Notably, a recent study that harmonized data from several SCD assessments (including the ECog) showed that the most robust items (with the highest information values) for high cognitive concern were almost exclusively related to memory problems.^43^ Other studies on AD biomarkers and SCD have suggested that amyloid beta (Aβ) deposition is associated with SCD‐related worries and heightened memory deficit awareness (i.e., hypernosognosia).^45^, ^46^ This would indicate that worries about self‐perceived decline may reflect an early symptom of Aβ pathology rather than subjective cognitive functioning. However, the association between SCD and Aβ deposition may vary by ethnic or racial group,^47^ and the link between SCD and accumulation of tau has also been noted. In the Harvard Aging Brain Study, SCD (using a version of the ECog) was reportedly indicative of accumulation of early pathology in the medial temporal lobe, and to a lesser extent, elevated global levels of Aβ.^48^ Because the medial temporal lobes are important for episodic memory, accumulation of pathology in this region may partially explain the association between subjective experiences of memory decline and more pronounced memory decline among those with cognitive concerns.

SCD may be an early indicator of ADRD when interventions may be most effective. In non‐Hispanic/Latino samples, SCD reporting has been identified as a possible early risk marker of cognitive decline that can help aid in the diagnosis of pre‐symptomatic AD.^2^, ^4^

In our study, 64% of cognitively unimpaired individuals had cognitive concerns. The associations between SCD with global and domain‐specific changes in cognitive performance were attenuated among the cognitively unimpaired subpopulation, except for the association between subjective memory decline and objective memory decline. Previous studies have reported mixed results regarding these associations. In the VETSA study, the associations of participant‐reported ECog scores and objective declines in memory and executive function were not statistically significant after excluding MCI cases.^40^ In a sample of cognitively healthy Mexican American older adults in the Health and Aging Brain Study–Health Disparities (HABS‐HD) study, subjective cognitive complaints, which were reported in 48% of the participants, were related to lower global and episodic memory scores.^49^ Of note, the HABS‐HD study only used one question to assign groups with and without subjective cognitive complaints and the VETSA study had a 39‐item assessment. Without a consistent measurement for SCD, other discrepancies in the results may also arise, but overall, our results support that the relationship between SCD and objective changes to cognition among cognitively healthy individuals may be driven by memory changes.

The population in this study is diverse in genetic ancestry, culture, social, and environmental exposures. Social processes may impact self‐perceived cognitive decline and objective cognitive decline reporting. Zlatar et al. previously reported significant and consistent differences in global and domain‐specific SCD (assessed by the ECog‐12) by Hispanic/Latino background or heritage groups,^8^ suggesting variability in SCD reporting among these groups. Examining this variability in SCD reporting is a limitation here, and a future direction. Another important question is whether race interacts with our exposures. Most of our sample, which includes ≈ 20% US‐born individuals, was born outside of the United States. The construct of race is complex and nuanced among Hispanic/Latino individuals, particularly among those who are foreign born. When asked about race, the majority of our sample could not respond to the question, refused, or reported more than one race (56%). Therefore, race reporting among Hispanics/Latinos in this large and representative cohort may not be meaningful, and we did not use race as a variable in our analysis.

There are some limitations to consider in this study. The ECog‐12 assessment measures cognitively mediated functional abilities rather than SCD directly.^19^ Participants in community‐based representative samples may report cognitive concerns or worries differently than in clinical settings.^4^ This study does not include dyadic SCD assessments, which may provide additional information about disease stage and progression. Despite the limitations, there are strengths to consider. ADRD studies would suggest that ADRD is progressive and develops over many years, and this large, well‐characterized community‐based population study examined objective 7‐year changes in cognitive performance based on participants’ subjective appraisals of cognitive decline.

We found that the subjective reports of cognitive decline were linked to objectively measured 7‐year cognitive decline in Hispanic/Latino middle‐aged and older adults living in the United States. The relationship between SCD and cognitive decline was stronger for men than women. The relationship between subjective memory decline and objective memory decline was stronger among those with cognitive concerns, and remained in cognitively healthy individuals, which may have implications for understanding the link between SCD and ADRDs. Further studies are needed to characterize SCD's neural and biomarker correlates in this population. This study underscores the importance of evaluating cognitive concerns among middle‐aged and older Hispanic/Latino adults during routine health‐care visits.

## CONFLICT OF INTEREST STATEMENT

The authors have no conflicts of interest to report and nothing to disclose. Author disclosures are available in the supporting information.

## CONSENT STATEMENT

HCHS/SOL and SOL‐INCA was approved by the institutional review boards at all participating institutions, and participants gave written informed consent.

## Supporting information

Supporting Information

Supporting Information
