# Supplementary material for: Subjective cognitive decline and cognitive change among diverse middle‐aged and older Hispanic/Latino adults: Results from the Study of Latinos–Investigation of Neurocognitive Aging (SOL‐INCA)
Source: Alzheimers Dement. 2024 Sep 5;20(11):7715–28. doi: 10.1002/alz.14232 (PMC11567848; doi:10.1002/alz.14232)
Supplement: Supplementary file 1 — Supporting Information [file ALZ-20-7715-s001.docx]

Supplemental Table 1. Means of cognitive scores by prevalent cognitive concern in the overall SOL-INCA population

|  |  | **No Cognitive Concern** | **Cognitive Concern** | **Overall** | **P-Value** |
| --- | --- | --- | --- | --- | --- |
|  |  | (N=2,048) | (N=4,177) | (N=6,225) |  |
|  |  | **Mean (SD)** | **Mean (SD)** | **Mean (SD)** |  |
| **Visit 1, Visit 2, and Change** | | |  |  |  |
|  | **B-SEVLT-Sum V1** | 23.52 (5.40) | 22.20 (5.73) | 22.64 (5.65) | **<0.001** |
|  | **B-SEVLT-Sum V2** | 23.62 (5.78) | 22.05 (5.93) | 22.57 (5.93) | **<0.001** |
|  | **B-SEVLT-Recall V1** | 8.54 (2.82) | 7.98 (2.93) | 8.17 (2.90) | **<0.001** |
|  | **B-SEVLT-Recall V2** | 8.77 (2.84) | 7.84 (3.08) | 8.15 (3.03) | **<0.001** |
|  | **WF V1** | 19.09 (7.04) | 18.37 (7.34) | 18.61 (7.24) | **0.005** |
|  | **WF V2** | 18.72 (7.22) | 17.75 (7.46) | 18.07 (7.39) | **0.001** |
|  | **DSS V1** | 35.81 (13.45) | 33.42 (13.13) | 34.22 (13.29) | **<0.001** |
|  | **DSS V2** | 33.81 (13.66) | 30.83 (13.19) | 31.82 (13.43) | **<0.001** |
|  | **Z-Score B-SEVLT Sum V2** | 0.19 (0.97) | -0.08 (1.00) | 0.01 (1.00) | **<0.001** |
|  | **Δ B-SEVLT Sum** | 0.11 (0.98) | -0.04 (1.00) | 0.01 (0.99) | **<0.001** |
|  | **Z-Score B-SEVLT Recall V2** | 0.22 (0.93) | -0.09 (1.01) | 0.01 (1.00) | **<0.001** |
|  | **Δ B-SEVLT Recall** | 0.16 (0.94) | -0.07 (1.02) | 0.01 (1.00) | **<0.001** |
|  | **Z-Score WF V2** | 0.10 (0.98) | -0.03 (1.01) | 0.01 (1.00) | **0.001** |
|  | **Δ WF** | 0.06 (0.98) | -0.03 (1.01) | 0.00 (1.00) | **0.021** |
|  | **Z-Score DSS V2** | 0.16 (1.02) | -0.06 (0.98) | 0.01 (1.00) | **<0.001** |
|  | **Δ DSS** | 0.09 (0.98) | -0.04 (1.01) | 0.00 (1.00) | **0.001** |
|  | **Z-Score Reversed Trails-A V2** | 0.11 (0.94) | -0.03 (1.00) | 0.02 (0.98) | **<0.001** |
|  | **Z-Score Reversed Trails-B V2** | 0.15 (0.99) | -0.06 (1.00) | 0.01 (1.00) | **<0.001** |
|  | **Z-Score Global Cognition V2** | 0.16 (0.76) | -0.07 (0.79) | 0.01 (0.79) | **<0.001** |
|  | **Δ Global** | 0.12 (0.95) | -0.05 (1.01) | 0.01 (0.99) | **<0.001** |

Results are derived from Wald tests using data from the Study of Latinos-Investigation of Neurocognitive Aging (SOL-INCA unweighted n = 6,225); Note 1: Sample size is unweighted; all other reported values are weighted; Note 2: SD=standard deviation, B-SEVLT= Brief-Spanish English Verbal Learning Test, WF=Word Fluency, DSS=Digit Symbol Substitution, Global=Global Cognition, Trials-A= Trail Making Test – Part A; Trails-B= Trail Making Test – Part B, Δ=change; Note 3: Bold values denote statistical significance

Supplemental Table 2. Descriptive characteristics by cognitive concern in the cognitively unimpaired subpopulation, excluding individuals with mild cognitive impairment or suspected severe impairment

|  |  | **No Cognitive Concern** | **Cognitive Concern** | **Overall** | **P-value** |
| --- | --- | --- | --- | --- | --- |
|  |  | (N=1,978) | (N=3,585) | (N=5,563) |  |
| **Age (%, SE)** | |  |  |  |  |
|  | <60 Years | 69.55 (1.55) | 65.76 (1.39) | 67.13 (1.07) | 0.209 |
|  | 60-69 Years | 25.05 (1.47) | 28.15 (1.22) | 27.02 (0.94) |  |
|  | 70+ Years | 5.40 (0.87) | 6.09 (0.70) | 5.84 (0.56) |  |
| **Sex (%, SE)** | |  |  |  |  |
|  | Female | 46.93 (1.51) | 58.63 (1.11) | 54.39 (0.88) | **<0.001** |
|  | Male | 53.07 (1.51) | 41.37 (1.11) | 45.61 (0.88) |  |
| **Education (%, SE)** | |  |  |  |  |
|  | Less than HS | 31.87 (1.59) | 39.65 (1.32) | 36.83 (1.08) | **<0.001** |
|  | HS or equivalent | 20.46 (1.26) | 22.02 (1.07) | 21.46 (0.81) |  |
|  | More than HS | 47.66 (1.51) | 38.33 (1.32) | 41.71 (1.04) |  |
| **Hispanic/ Latino background (%, SE)** | |  |  |  |  |
|  | Dominican | 11.38 (1.15) | 8.21 (0.76) | 9.36 (0.77) | **0.006** |
|  | Central American | 6.79 (0.78) | 7.53 (0.67) | 7.26 (0.54) |  |
|  | Cuban | 24.88 (2.24) | 26.38 (2.16) | 25.84 (1.98) |  |
|  | Mexican | 32.36 (2.09) | 34.45 (1.89) | 33.69 (1.73) |  |
|  | Puerto-Rican | 14.24 (1.03) | 15.30 (0.97) | 14.92 (0.82) |  |
|  | South American | 5.14 (0.57) | 5.28 (0.51) | 5.23 (0.42) |  |
|  | More than one/Other | 5.22 (0.95) | 2.84 (0.49) | 3.70 (0.47) |  |
| **Age (Mean, SD)** | | 55.46 (7.65) | 56.27 (8.12) | 55.98 (7.96) | **0.031** |
| **Framingham CVD 10-year risk (Mean, SD)** | | 15.63 (13.10) | 15.10 (13.04) | 15.29 (13.07) | 0.363 |
| **CESD-10 Depressive symptoms (Mean, SD)** | | 5.41 (5.05) | 8.14 (6.60) | 7.15 (6.22) | **<0.001** |
| **10-Item State Trait Anxiety Inventory (Mean, SD)** | | 14.97 (4.79) | 17.63 (6.01) | 16.67 (5.74) | **<0.001** |

Results are derived from survey linear regression models using data from the cognitively unimpaired subsample in the Study of Latinos-Investigation of Neurocognitive Aging (SOL-INCA unweighted n = 5,563); Note 1: Sample size is unweighted; all other reported values are weighted; Note 2: All variables are measured at Visit 1; Note 3: SE=standard error, SD=standard deviation, HS=high school, CVD=cardiovascular disease, CESD= Center for Epidemiological Studies-Depression; Note 4: Bold values denote statistical significance**S**UPPLEMENTAL FIGURES

**Supplemental Figure 1.** Association between ECog-12 executive and change in cognitive performance in the overall SOL-INCA population

**
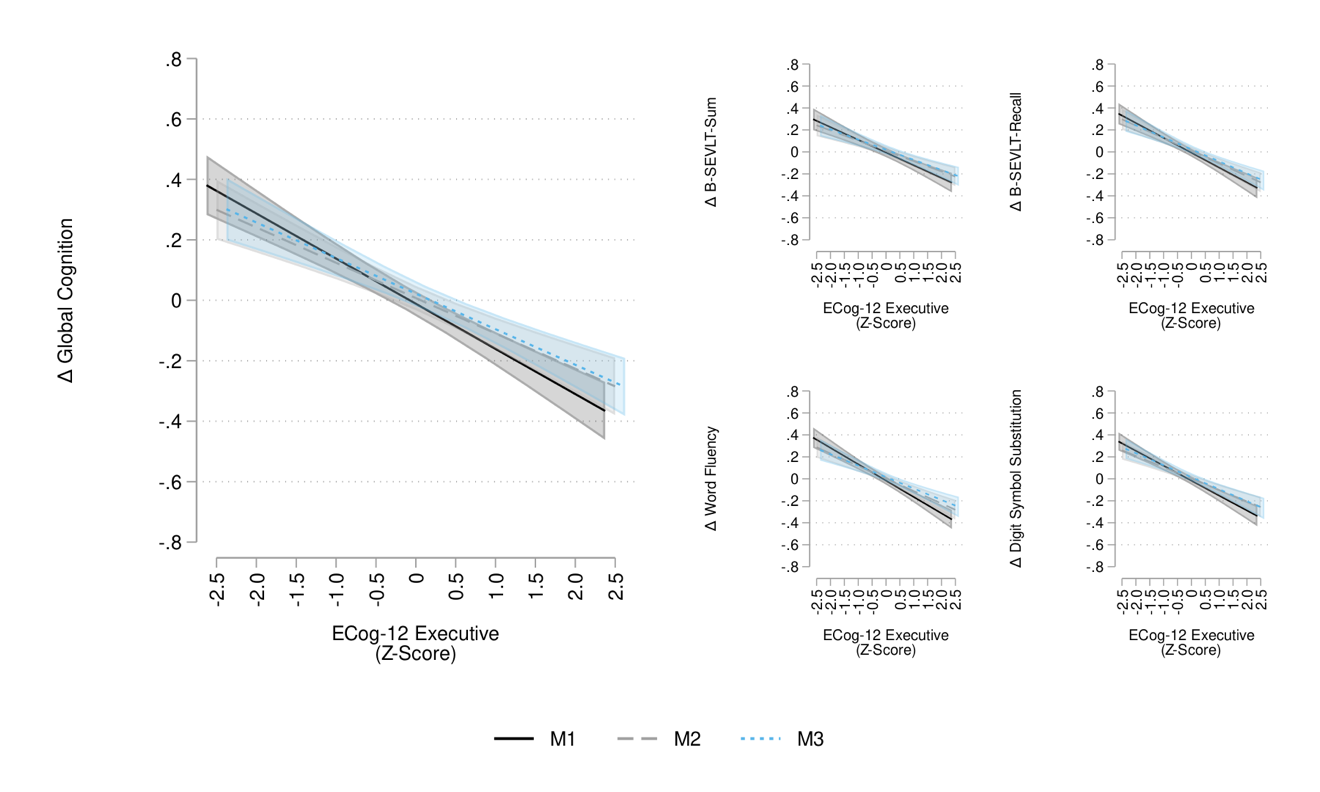
**

Results are derived from survey linear regression models using data from the Study of Latinos-Investigation of Neurocognitive Aging (SOL-INCA unweighted n = 6,225); Note 1: ECog-12 = 12-Item form of the Everyday Cognition Scale, B-SEVLT= Brief-Spanish English Verbal Learning Test, Executive = Executive Function, Δ=change; Note 3: Model 1 is a unadjusted model, Model 2 is adjusted for age, sex, education, and Hispanic/Latino background, Model 3 is additionally adjusted for Framingham Cardiovascular Disease 10-year risk score, Center for Epidemiological Studies-Depression - Depressive Symptoms, and 10-Item State Trait Anxiety Inventory

**Supplemental Figure 2.** Association between ECog-12 memory and change in cognitive performance in the overall SOL-INCA population


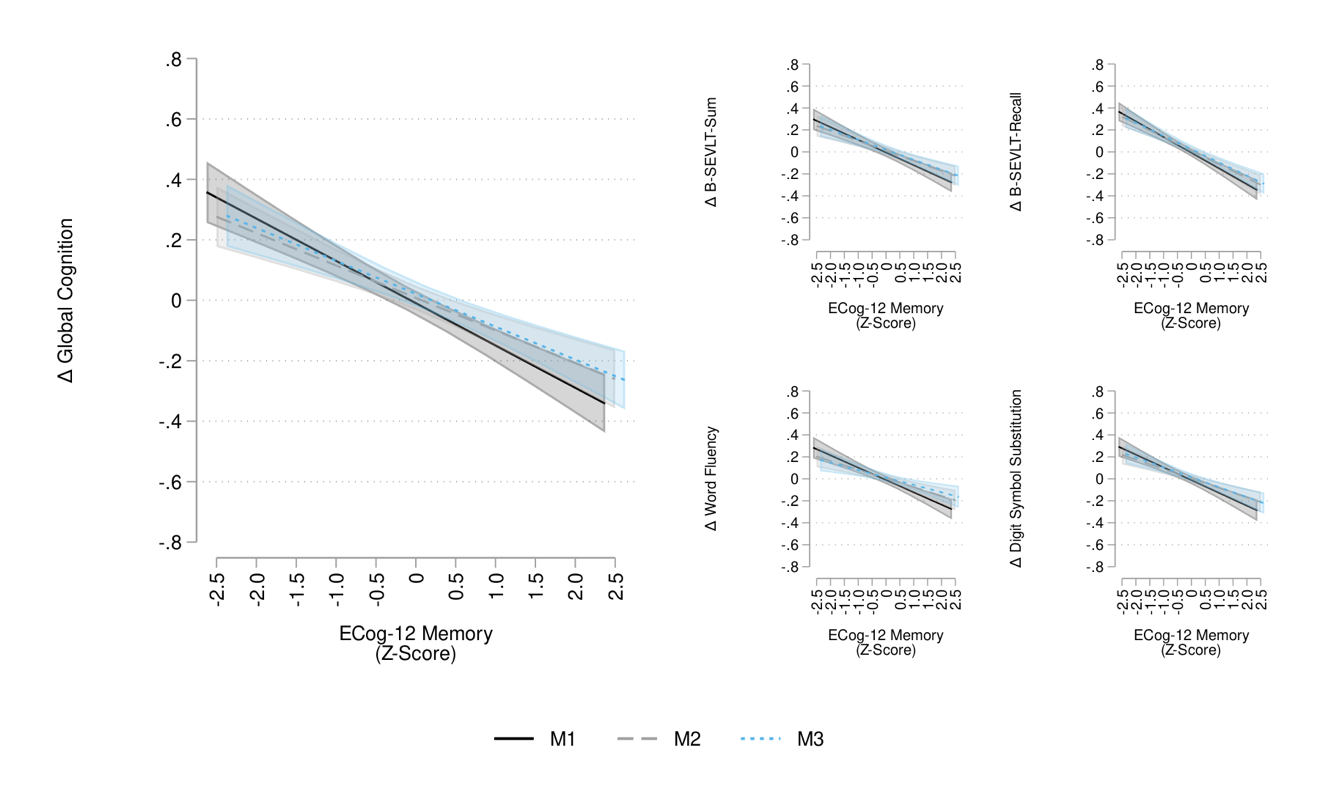


Results are derived from survey linear regression models using data from the Study of Latinos-Investigation of Neurocognitive Aging (SOL-INCA unweighted n = 6,225); Note 1: ECog-12 = 12-Item form of the Everyday Cognition Scale, B-SEVLT= Brief-Spanish English Verbal Learning Test, Δ=change; Note 3: Model 1 is a unadjusted model, Model 2 is adjusted for age, sex, education, and Hispanic/Latino background, Model 3 is additionally adjusted for Framingham Cardiovascular Disease 10-year risk score, Center for Epidemiological Studies-Depression - Depressive Symptoms, and 10-Item State Trait Anxiety Inventory

**Supplemental Figure 3.** Association between ECog-12 and change in cognitive performance in the cognitively unimpaired subpopulation, excluding individuals with mild cognitive impairment or suspected severe impairment


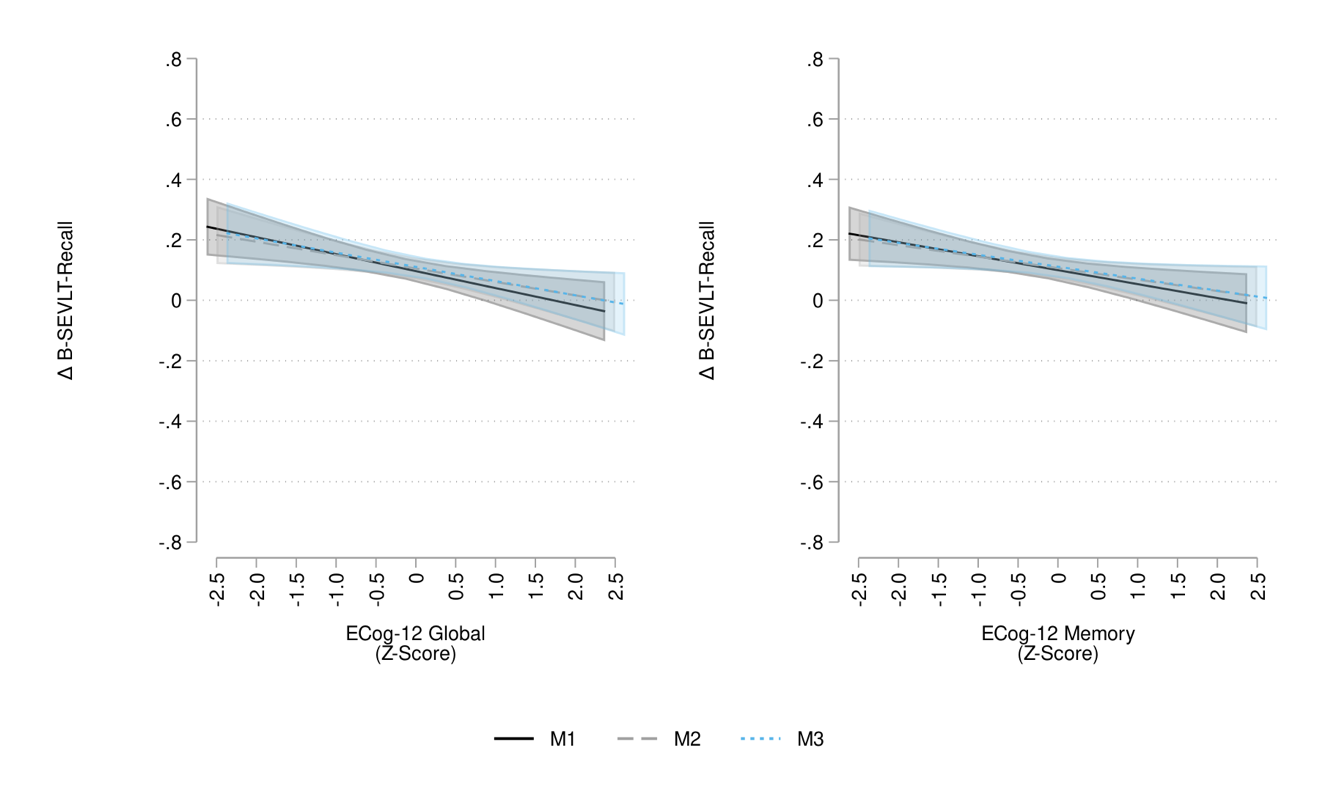


Results are derived from survey linear regression models using data from the cognitively unimpaired subsample in the Study of Latinos-Investigation of Neurocognitive Aging (SOL-INCA unweighted n = 5,563); Note 1: ECog-12 = 12-Item form of the Everyday Cognition Scale, B-SEVLT= Brief-Spanish English Verbal Learning Test, Global= Global Cognition, Δ=change; Note 3: Model 1 is a unadjusted model, Model 2 is adjusted for age, sex, education, and Hispanic/Latino background, Model 3 is additionally adjusted for Framingham Cardiovascular Disease 10-year risk score, Center for Epidemiological Studies-Depression - Depressive Symptoms, and 10-Item State Trait Anxiety Inventory
